# Clinical efficacy of hyperbaric oxygen combined with different timings of right median‐nerve electrical stimulation in patients with brain injury‐induced disorders of consciousness

**DOI:** 10.1002/brb3.2716

**Published:** 2022-08-03

**Authors:** Yan‐Song Liu, Zi‐bo Liu, Zhe Yang, Long Zhao, Hong‐Ling Li

**Affiliations:** ^1^ The Scond Department of Rehabilitation The Second Hospital of Hebei Medical University Shijiazhuang China; ^2^ Department of Endocrinology The Second Hospital of Hebei Medical University Shijiazhuang China

**Keywords:** brain injury, disorders of consciousness, hyperbaric oxygen therapy, right median‐nerve electrical stimulation

## Abstract

**Objective:**

The objective of this study was to investigate the clinical efficacy of hyperbaric oxygen combined with right median‐nerve stimulation (RMNES) in patients with disorders of consciousness caused by brain injury.

**Methods:**

A total of 120 patients with consciousness disorders caused by brain injury were selected. They were randomly divided into three groups, a control group, test group 1 (treated with RMNES after hyperbaric oxygen therapy [HBOT]), and test group 2 (treated with RMNES at the same time as HBOT), with 40 patients in each group. Before and after treatment, the Glasgow coma scale (GCS), brainstem auditory‐evoked potential (BAEP), electroencephalogram (EEG), and upper‐limb sensory‐evoked potential (USEP) were evaluated for the three groups of patients.

**Results:**

The GCS score of patients in the three groups significantly improved compared with that before treatment (*p* < .05). There were significant differences in GCS scores among the three groups (*p* < .05), and the GCS score for the patients was test group 2>test group 1>control group. The EEG, BAEP, and USEP scores were significantly improved compared with those before treatment (*p* < .05), and the degree of improvement of patients in the three groups was test group 2>test group 1>control group (*p* < .05). The clinical efficacy of test group 2 was higher than that of test group 1, and the clinical efficacy of test group 1 was higher than that of the control group (*p* < .05).

**Conclusion:**

Hyperbaric oxygen combined with RMNES can improve the state of consciousness and promote the recovery of consciousness for patients with consciousness disorders caused by brain injury, and the effect of RMNES combined with HBOT in the chamber on improving the recovery of consciousness is better than after HBOT outside the chamber.

## Graphical abstract

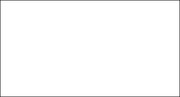

## 1 INTRODUCTION

Disorder of consciousness (DOC) (Edlow et al., 2021) is a condition in which the patient's ability to perceive environmental stimuli is reduced or lost to varying degrees. DOC is usually caused by severe brain injury, which can lead to coma, minimally conscious states (MCS), and unresponsive wakefulness syndrome (UWS) (Hermann et al., 2019). UWS is also known as the vegetative state (VS). Patients with UWS exhibit arousal fluctuations but have no consciousness, while patients with MCS (Giacino et al., 2002; Giacino, Katz, Schiff, Whyte, et al., 2018) are characterized by the presence of the faintest, but clearly present, self‐awareness or environmental awareness.

The causes of DOC are complex and include neurological diseases, poisoning, shock, heart failure, and various accidental craniocerebral injuries. The disease develops rapidly with high mortality and disability rates, thus placing a heavy burden on families and society. Foreign epidemiological studies have shown that the incidence of DOC after brain injury ranges from 0.5% to 1.8% (Moattari et al., 2016). In China, there are approximately 2 million patients with DOC caused by various types of brain injury every year (Giacino, Katz, Schiff, John, et al., 2018). Although the rapid development of modern medicine has greatly reduced the mortality rate of patients with major diseases, this progress has been accompanied by an increase in the number of patients with DOC. Moreover, these patients are bedridden for a long time, and their quality of life is greatly reduced.

The current methods for promoting the recovery of consciousness in patients with DOC include (D. X. Jiang et al., 2016; Morgan et al., 2012; Thibaut et al., 2019) basic nursing treatment (D. X. Jiang et al., 2016), drug awakening (D. X. Jiang et al., 2016; Morgan et al., 2012; Thibaut et al., 2019), surgical treatment (Thibaut et al., 2019), various sensory and nerve electrical stimulation therapies (Thibaut et al., 2019), traditional Chinese medicine treatments such as acupuncture and massage (D. X. Jiang et al., 2016), hyperbaric oxygen therapy (HBOT) (Lin et al., 2008), mild hypothermia therapy, and neural stem‐cell therapy (D. X. Jiang et al., 2016). Although there are many treatment methods for patients with DOC, the outcomes are still unsatisfactory.

HBOT (Lin et al., 2008) has been used as a noninvasive treatment for the recovery of consciousness in patients with traumatic brain injury, stroke, and postcardiopulmonary resuscitation with some success.

Median‐nerve electrical stimulation (MNES) is a treatment method that uses low‐frequency electricity to electrically stimulate the skin of the anatomical area where the median nerve is located on the ventral side of the patient's wrist or forearm (Shi et al., 2017). Since 1996, it has been used to promote wakefulness in patients with DOC (Yokoyama et al., 1996), and its application has become gradually popularized because it is inexpensive, noninvasive, and easy to perform (Shi et al., 2017); however, there have been few relevant studies in China. After reviewing the relevant literature (Ruan et al., 2019; Tao et al., 2019; Zhang et al., 2010), it was found that most of the current studies only investigated the therapeutic effect of RMNES on disturbance of consciousness alone and did not explore whether there is a better treatment to help the recovery of disturbance of consciousness in combination with other treatments. Therefore, the novelty of this study is to combine these two treatment methods, explore the clinical efficacy of right MNES (RMNES) combined with HBOT in the treatment of DOC caused by brain injury at different times, and explore whether the effect of the combination of the two methods is better than that of single treatment, providing new ideas for clinical diagnosis and treatment.

## 2 MATERIALS AND METHODS

### 2.1 Subjects

A total of 120 patients with DOC who were admitted to the Second Hospital of Hebei Medical University between December 2017 and June 2020 with a clear diagnosis and met the inclusion criteria were selected for this study. The diagnosis of consciousness disorder was based on the Glasgow scale, and patients with consciousness disorder were diagnosed with a score of less than 8.

### 2.2 Inclusion and exclusion criteria

Inclusion criteria were as follows: (1) causes of brain injury: traumatic brain injury, cerebral hemorrhage, cerebral infarction and postcardiopulmonary resuscitation; (2) degree of impaired consciousness: 3 points <Glasgow coma scale (GCS) score ≤ 8 points; (3) stable respiratory and circulatory system indexes; (4) 18 ≤ age ≤ 70 years; (5) first episode of the disease with a duration of less than 1 month; and (6) the informed consent and signature of the patient's family.

Exclusion criteria were as follows: (1) patients with unstable basic vital signs, such as heart rate, blood pressure, and respiration; (2) a previous history of epilepsy or frequent seizures; (3) pregnant women; (4) patients with severe cardiac arrhythmias or pacemaker implants; (5) patients with steel nails or plates implanted at the treatment site; (6) patients who were clinically assessed to be unsuitable for trials using electrical stimulation products; and (7) patients with multiple organ dysfunction.

### 2.3 Grouping

Using the random number table method, 120 patients who met the criteria were randomly divided into a control group, test group 1, and test group 2, with 40 patients in each group. That is, the patients were numbered in sequence according to the order of admission, and then the patients were grouped according to the order in the random number table, and the numbers exceeding 120 in the random number table were removed. As shown in Table 1, there was no difference in age, sex, duration, or type of injury between these three groups (*p* > .05).

**TABLE 1:** Comparison of the basic conditions of the three groups of patients before treatment

			Gender			Causes of disease	
Group	Number of cases	Age (years)	Male	Female	Duration of illness (days)	Traumatic brain injury	Nontraumatic brain injury
Test group 1	40	42.15 ± 14.15	22	18	16.65 ± 1.33	20	20
Test group 2	40	42.5 ± 13.78	24	16	16.85 ± 1.45	21	19
Control group	40	41 ± 14	20	20	17.4 ± 1.55	21	19

### 2.4 Treatment methods

#### 2.4.1 Control group

*Routine care and treatment*: All patients were given blood pressure, electrocardiogram, and oxygen saturation monitoring on the ward. Corresponding medication and surgical treatment were administered according to the changes in the patients' conditions. Measures such as the regulation of water and electrolyte balance, anti‐infection treatment, dehydration and intracranial pressure reduction, and nutritional support treatment were taken.

*HBOT protocol*: The therapeutic pressure was 2.0 ATA, the hyperbaric oxygen chamber was pressurized from 1.0 ATA, and the pressure was stabilized after pressurization to 2.0 ATA, with a pressurization time of 25 min. After pressurization, pure oxygen was inhaled twice for 30 min, and air was inhaled at intervals of 10 min. This was followed by decompression, with a pressure reduction from 2.0 ATA to 1.0 ATA and a decompression time of 25 min. This treatment was administered once a day, with a 1‐week course of treatment.

*Rehabilitation treatment plan*: (1) Cerebellar fastigial nucleus electrical stimulation: the electrode was applied behind the patient's ear to improve the posterior circulation of the patient's brain, and the stimulation intensity was the normal tolerance amount of approximately 15 mA; (2) limb electrical stimulation: electrodes were applied to the radial carpal extensor and tibialis anterior muscle belly of the patient's limb with a stimulation intensity that was appropriate for achieving maximum muscle contraction without the patient experiencing pain; patients with no obvious movement of the limbs were subjected to electrical stimulation of the limbs, and patients with hemiplegia were subjected to electrical stimulation of the affected limb; (3) passive activity of the limb at the bedside; (4) VitalStim electrical stimulation: the stimulation site was in the swallowing muscle group, and this treatment was aimed at preventing atrophy in this group. The abovementioned conventional rehabilitation treatment was performed once a day for 30 min, six times a week, with a 1‐week course of treatment.

#### 2.4.2 Test group

This group was divided into test group 1 and test group 2, and both were treated with RMNES on the same basis as the control group. Test group 1 received RMNES treatment after HBOT, and test group 2 received RMNES treatment in the chamber at the same time as HBOT. The treatment site was 2 cm above the right transverse wrist, and the two electrode pads were placed side by side; the output stimulation waveform was a unidirectional square wave, the treatment frequency was 40 Hz, the pulse width was 300 ms, the current intensity was 1−40 mA, and the stimulation intensity was based on the observation of flexion and twitching of the right middle finger, which was generally 10−20 mA. This treatment was performed every 2 h, once a day, with a 1‐week course of treatment.

### 2.5 Assessment methods

The GCS, electroencephalogram (EEG), upper‐limb sensory‐evoked potential (USEP), brainstem auditory‐evoked potential (BAEP), and clinical outcomes were evaluated before the treatment and after the four treatment sessions.

1. The GCS scale (Kebapçı et al., 2020) has three components: eye opening response (maximum 4 points), verbal response (maximum 5 points), and motor response (maximum 6 points). A GCS score of 15 indicated that the patient was clearly conscious, a score of 12−14 indicated that the patient was in a state of mild DOC, a score of 9−11 indicated that the patient was in a state of moderate DOC, and a score of 8 or less indicated that the patient was in a severe state of DOC.
2. The EEG assessment was performed using 16 leads with disk electrodes placed according to the international 10/20 system standard, and the grading criteria followed Hockaday et al.’s grading of DOC (Hockaday et al., 1965). Grade I patients had near‐normal EEG waves with the basic rhythm of α waves and a score of 3; grade II patients had predominantly θ waves with a few δ waves and a score of 2; grade III patients had predominantly δ waves with no other rhythmic activity and a score of 1; grade IV indicated that all waves in the patient's EEG were absent and had a score of 0.
3. The BAEP assessment was performed according to the international 10/20 system of electrode placement, and the grading standard was based on the Greenberg criteria (Greenberg et al., 1977). The waveforms of grade I patients were basically normal, with a score of 3; the I–V waves of grade II patients were clear and distinguishable, but the latency was prolonged, and the amplitude was decreased, with a score of 2; the latency and amplitude of the I wave of grade III patients were normal, and some of the remaining waves were present or showed indistinct positive phase waves, with a score of 1; the waveforms of grade IV patients were difficult to distinguish, or only the I wave was present, with a score of 0.
4. The USEP assessment was performed by placing electrodes according to the international 10/20 system, and the grading standard referred to the Greenberg criteria (Greenberg et al., 1977). Grade I patients had basically normal waveforms with a score of 3; grade II patients had missing waveform components after 50 ms, reduced amplitude and prolonged latency, with a score of 2; grade III patients had only P15 and N20 waves and missing waveform components after 20 ms, with a score of 1; and in grade IV, there were no waves present or only P15 waves, with a score of 0.
5. Criteria for the clinical efficacy assessment included the following (Tian et al., 2012):
6. Basically cured: the patient was awake and had a GCS score of 15.
7. Significantly effective: significant improvement in signs and symptoms and a GCS score >12.
8. Effective: improvement in signs and symptoms and a GCS score >9.
9. Ineffective: no improvement in signs or symptoms, no improvement or a decrease in the GCS score.
$$\begin{array}{ccc} \mathrm{Effective}\;\mathrm{rate} & = & \left[\left(\mathrm{basically}\;\mathrm{cured}+\mathrm{significantly}\;\mathrm{effective}+\mathrm{effective}\right)/\right.\\ & & \;\;\left.\mathrm{number}\;\mathrm{of}\;\mathrm{patients}\;\mathrm{treated}\right]\times 100\%. \end{array}$$
10. Security assessment: Patients were observed for adverse effects such as seizures, skin burns at the MNES site, and sympathetic excitation, which had been reported in previous trials during treatment.
11. Follow‐up visit: All patients were followed up for 6 months by outpatient follow‐up or telephone interview. If the patient and his/her relatives were not available, the patient was recorded as lost to follow‐up. The patient's outcome at the 6‐month follow‐up was defined as recovery of consciousness, MCS, UWS, or death. The patients' families were followed up by telephone, and the patients were scored by the Glasgow prognostic score (GPS). The criteria for the recovery of consciousness were clear consciousness, the ability to functionally communicate with others, the ability to correctly identify the figure, time and place, and the ability to correctly perform motor commands. The criteria for MCS were the ability to perform visual tracking, the ability to perform directional voluntary movements, and the ability to localize pain but not to functionally communicate with others. VS was diagnosed when the patient had complete loss of cognition of the self and peripheral environment, no conscious activity, and inability to understand or express language, but there was a sleep–wake cycle and an ability to maintain voluntary breathing and blood pressure.

## 3 STATISTICAL ANALYSIS METHODS

The trial results were processed by statistical analysis using SPSS 21.0 software. Measurement data were first tested for normality and homogeneity of variance. If normality and homogeneity of variance were observed, the *t*‐test was performed, and the results were expressed as the mean ± standard deviation ($\bar{x}$ ± s); if non‐normality and/or heterogeneity of variance was observed, a nonparametric test was applied. The chi‐square test was used for the comparison of percentages. The test level was *α* = 0.05, and *p* < .05 was considered a statistically significant difference.

## 4 RESULTS

### 4.1 GCS score

Before treatment, there was no difference in GCS scores among the three groups (*p* > .05); after treatment, GCS scores improved in all three groups (*p* < .05), and the scores were compared as follows: test group 2 > test group 1 > control group (*p* < .05; Table 2).

**TABLE 2:** Glasgow coma scale (GCS) scale scores of patients in the three groups ($\bar{x}$ ± s, *n* = 40 in test group 1, *n* = 40 in test group 2, and *n* = 40 in the control group)

Group	Before treatment	After treatment
Test group 1	5.35 ± 1.66^a^	7.75 ± 1.48^b^, ^c^
Test group 2	5.45 ± 1.7^a^	9.35 ± 1.53^b^, ^c^
Control group	5.65 ± 1.57^a^	6.75 ± 0.34^b^, ^c^

^a^ *p* > .05 for a two‐by‐two comparison between groups before treatment.

^b^ *p* < .05 for a two‐by‐two comparison between groups after treatment.

^c^ *p* < .05 for the comparison of the three groups before and after treatment.

### 4.2 EEG score

Before treatment, the overall score for test group 1 was 1.00 ± 0.85, the score for test group 2 was 1.05 ± 0.88, and the score for the control group was 1.20 ± 0.82. There was no statistically significant difference between the three groups before treatment (*p* > .05). After treatment, the scores were 2.00 ± 0.75 in test group 1, 2.375 ± 0.77 in test group 2, and 1.50 ± 0.17 in the control group. All three groups had improved scores after treatment compared with those before treatment, and the difference was statistically significant (*p* < .05). The EEG scores in test group 2 were better than those in test group 1 and the control group after treatment (*p* < .05; Table 3).

**TABLE 3:** Comparison of electroencephalogram (EEG) scores among the three groups of patients ($\bar{x}$ ± s, *n* = 40 in test group 1, *n* = 40 in test group 2, and *n* = 40 in the control group)

Group	Before treatment	After treatment
Control group	1.20 ± 0.82^a^	1.50 ± 0.17^b^, ^c^
Test group 1	1.00 ± 0.85^a^	2.00 ± 0.75^b^, ^c^
Test group 2	1.05 ± 0.88^a^	2.375 ± 0.77^b^, ^c^

^a^ *p* > .05 for a two‐by‐two comparison between groups before treatment.

^b^ *p* < .05 for a two‐by‐two comparison between groups after treatment.

^c^ *p* < .05 for the comparison of the three groups before and after treatment.

### 4.3 BAEP Score

Before treatment, the overall score for test group 1 was 1.20 ± 0.61, test group 2 was 1.20 ± 0.61, and the control group was 1.25 ± 0.63. There was no statistically significant difference between the three groups before treatment (*p* > .05). After treatment, the overall score was 2.00 ± 0.78 in test group 1, 2.50 ± 0.64 in test group 2, and 1.53 ± 0.78 in the control group. The scores of the three groups were improved after treatment compared with before treatment, and the difference was statistically significant (*p* < .05). The BAEP score was better in test group 2 than in test group 1 and the control group after treatment (*p* < .05; Table 4).

**TABLE 4:** Comparison of brainstem auditory‐evoked potential (BAEP) scores among the three groups of patients ($\bar{x}$ ± s, *n* = 40 in test group 1, *n* = 40 in test group 2, and *n* = 40 in the control group)

Group	Before treatment	After treatment
Control group	1.25 ± 0.63^a^	1.53 ± 0.78^b^, ^c^
Test group 1	1.20 ± 0.61^a^	2.00 ± 0.78^b^, ^c^
Test group 2	1.20 ± 0.61^a^	2.50 ± 0.64^b^, ^c^

^a^ *p* > .05 for a two‐by‐two comparison between groups before treatment.

^b^ *p* < .05 for a two‐by‐two comparison between groups after treatment.

^c^ *p* < .05 for the comparison of the three groups before and after treatment.

### 4.4 USEP

Before treatment, the overall score was 1.05 ± 0.68 in test group 1, 1.05 ± 0.68 in test group 2, and 1.13 ± 0.69 in the control group, with no statistically significant difference between the three groups before treatment (*p* > .05). After treatment, the overall score was 1.95 ± 0.60 in test group 1, 2.35 ± 0.48 in test group 2, and 1.55 ± 0.78 in the control group. The scores of the three groups were improved statistically after treatment compared with those before treatment (*p* < .05). The USEP score was better in test group 2 than in test group 1 and the control group after treatment (*p* < .05; Table 5).

**TABLE 5:** Comparison of upper‐limb sensory‐evoked potential (USEP) scores among the three groups ($\bar{x}$ ± s, *n* = 40 in test group 1, *n* = 40 in test group 2, and *n* = 40 in the control group)

Group	Before treatment	After treatment
Control group	1.13 ± 0.69^a^	1.55 ± 0.78^b^, ^c^
Test group 1	1.05 ± 0.68^a^	1.95 ± 0.60^b^, ^c^
Test group 2	1.05 ± 0.68^a^	2.35 ± 0.48^b^, ^c^

^a^ *p* > .05 for a two‐by‐two comparison between groups before treatment.

^b^ *p* < .05 for a two‐by‐two comparison between groups after treatment.

^c^ *p* < .05 for the comparison of the three groups before and after treatment.

### 4.5 Clinical efficacy assessment

As shown in Table 6, in this study, the overall response rate was 75% (30/40) in the control group, 80% (32/40) in test group 1, and 90% (36/40) in test group 2. The difference between the test group and the control group was statistically significant (*p* < .05), and the overall response rate was test group 2 > test group 1 > control group (*p* < .05).

**TABLE 6:** Comparison of clinical efficacy of patients in the three groups (cases %) (*n* = 40 in test group 1, *n* = 40 in test group 2, and *n* = 40 in the control group)

Group	Basically cured	Significantly effective	Effective	Ineffective	Overall response rate (%)
Test group 1	8	10	14	8	80
Test group 2	10	14	12	4	90
Control group	5	8	17	10	75

### 4.6 Follow‐up visits

Of the total number of patients, 77 (64.2%) patients were contacted for follow‐up. The results are shown in Table 7. A total of 26 patients were followed up in test group 2, of whom 14 (53.9%) regained consciousness, six (23.1%) were in MCS, and six (23.1%) were diagnosed with VS. A total of 26 patients were followed up in test group 1, of whom nine (34.6%) regained consciousness, six (23%) were in MCS, and 11 (42.3%) were diagnosed with VS. In the control group, 25 patients were followed up, of whom five (20%) regained consciousness, five (20%) were in MCS, and 15 (60%) were diagnosed with VS. The statistical results showed that the consciousness rate of patients in test group 2 was significantly higher than that in test group 1 and the control group (*p* < .05), and the number of patients diagnosed with VS was less than that in test group 1 and the control group, and the difference was statistically significant (*p* < .05). The GPS scores of all the patients at follow‐up showed that those in test group 2 were significantly higher than those in test group 2 and test group 1 (3.5 ± 1.3 vs. 3.0 ± 1.2 vs. 2.64 ± 0.99, *p* < .05).

**TABLE 7:** Patient follow‐up data for the three groups (cases) (*n* = 26 in test group 1, *n* = 26 in test group 2, and *n* = 25 in the control group)

State of Consciousness	Control group	Test group 1	Test group 2
Restore consciousness	5	9	14
MCS	5	6	6
VS	15	11	6

Abbreviations: MCS, minimally conscious states; VS, vegetative state.

### 4.7 Adverse reactions

During the trial, patients did not experience adverse effects such as seizures, skin burns at the MNES site, or sympathetic excitation, as reported in previous studies.

## 5 DISCUSSION

At present, the clinical treatment for the recovery of consciousness in DOC patients is based on comprehensive treatment with multidisciplinary cooperation. Although there are many treatment methods, the treatment results have their own advantages and disadvantages and are not ideal.

HBOT (Gonzales‐Portillo et al., 2019) is recognized for its significant effect on carbon‐monoxide poisoning, decompression sickness, air embolism, and ischemic‐hypoxic diseases. It is also effective in promoting the recovery of consciousness in DOC patients caused by traumatic brain injury, stroke, and postcardiopulmonary resuscitation. The theoretical basis of HBOT to promote recovery of consciousness is (Bennett et al., 2012; Gonzales‐Portillo et al., 2019; Tal et al., 2017; Sankaran et al., 2019; Ye et al., 2020) that it can increase the effective diffusion distance of blood oxygen and increase the amount of oxygen diffusion, as well as promoting vasoconstriction to speed up blood flow and accelerating the establishment of collateral circulation. At the same time, it can also increase blood flow in the ischemic area and vertebral artery and the ascending reticular activation system increase the production of ATP in brain cells and the synthesis of other substances in brain tissue and promote the recovery of brain function. The results of this study showed that the GCS score, EEG score, BAEP score, and USEP score improved in the control group at the end of HBOT combined with conventional clinical therapies and rehabilitation, which supports the above theory. The results of a study by Li and Liu (2019) on 120 patients with severe traumatic coma treated with HBOT showed that compared with the control group, the GCS score and brain function score of patients in the hyperbaric oxygen treatment group were improved. This is consistent with the results of this study.

MNES was introduced in 1996 for the treatment of comatose patients (Yokoyama et al., 1996). This stimulation method produces a therapeutic effect by sending signals generated by peripheral‐nerve electrical stimulation through the spinal cord to the brainstem, thalamus, and, ultimately, to the sensory‐motor areas corresponding to the hand in the cerebral cortex (J. Y. Jiang et al., 1988). Since hand function is localized to the largest area of cortical projections (Cooper et al., 2006), stimulation of the median nerve can achieve the greatest therapeutic effect. The mechanism of action may be related to increased cerebral blood perfusion, direct excitation of the cerebral cortex, and the increased secretion of the corresponding neurotransmitters.

In a functional magnetic resonance imaging study of the effects of RMNES on brain function in healthy subjects, M. Chen et al. (2019) found that the brain areas being activated in 28 subjects under RMNES were mainly concentrated in the hand‐motor and sensory functional areas of the left brain, including the precentral gyrus (BA4) where the primary motor cortex (M1) is located, the precentral gyrus (BA6) where the cortical premotor cortex (PMC) is located, the postcentral gyrus (BA2, BA3) where the primary somatosensory cortex (S1) is located, the supramarginal gyrus (BA40) where the secondary somatosensory cortex (S2) is located, and the left insula (BA13). Activation areas are also present in the right brain, mainly in the supramarginal gyrus where S2 is located and in the postcentral gyrus where S1 is located. Compared with the resting state, the BOLD signal intensity of the above activated brain regions was more variable in the RMNES wake‐promoting mode, with *T* > 5.84 and *p* < .05 (FWE corrected).

The results of Zhao and Liu's study (2020) on the effect of RMNES on DOC due to traumatic craniocerebral injury showed that the test group had relatively high levels of norepinephrine (NE) and dopamine and significantly decreased levels of β‐EP after RMNES, suggesting that RMNES can regulate the secretion of neurotransmitters in the body and may be one of the mechanisms that promote wakefulness.

It was also found that GABA contributed to the maintenance of the sleep state (M. Chen et al., 2019; Cy et al., 2016; Zhao & Liu, 2020), and the absence of its receptor, GABA‐b, delayed sleep time. The results of Wei et al.’s (Zhao & Liu, 2020; Cy et al., 2016; Vienne et al., 2010) study on the effect of MNES on the changes in GABA‐b expression in comatose rats after traumatic brain injury showed that the wake‐promoting effect of MNES on comatose rats was related to GABA‐b receptors.

The neuronal synapses of the median nerve are directly involved in ascending reticular activating system activity, which consists of three major arousal systems: the cholinergic system, the raphe dorsal nucleus‐serotonin system, and the locus coeruleus (LC)–noradrenergic system in which the LC synthesizes and secretes NE to innervate the entire cortex, diencephalon, and other regions of the brain. NE acts on the cerebral cortex through the excitatory receptor α1 to maintain the electrical physiological activity of the cerebral cortex and the waking state. Wei and Feng (2016) found that the pro‐wake effect of MNES in comatose rats after traumatic brain injury was associated with the upregulation of orexin‐A and the expression of its receptor, OX1R, in the prefrontal cortex (PFC). The effect of MNES on the expression of PFC NE α1 receptors in comatose rats after traumatic brain injury found by Feng et al. (2015) suggested that the mechanism of the pro‐wake effect of MNES on comatose rats after traumatic brain injury might be related to the increase in α1R expression in the PFC region. It was also confirmed that OX1R and its receptor OX1R were involved in the wakefulness‐promoting process of MNES, and the mechanism may be related to the upregulation of α1R expression in the PFC region by OX1R. In conclusion, the results of a series of studies suggest that MNES can regulate the levels of neurotransmitters associated with wakefulness.

Although it has been reported in the literature that electrical stimulation of both the left and right median nerves is effective in restoring consciousness impairment, the results of a study by Yang (2005) highlighted that in patients whose native language is Chinese, the dominant hemisphere in nonright‐handed people is still the left cerebral hemisphere and very rarely the right cerebral hemisphere.

Therefore, stimulation of the right median nerve is mostly chosen for clinical treatment.

In this study, the right median‐nerve region was selected for electrical stimulation treatment, and the test results showed that the GCS score, EEG score, BAEP score, USEP score, and clinical outcomes of patients in all three groups were significantly improved compared with those before treatment (*p* < .05). However, patients in test groups 1 and 2 showed much improvement than the control group, indicating that HBOT combined with RMNES treatment had a more significant wake‐promoting effect on patients with DOC. This may be related to the synergistic effect of RMNES and HBOT on cerebral blood flow, blood‐flow velocity, and neurotransmitter regulation. The improvement in scores from various assessments in test group 2, especially electrophysiological indexes, was better than that in test group 1, probably because RMNES was performed at the same time as HBOT, which enhanced the effect of the two treatments on the mechanism of wakefulness promotion of DOC; in test group 1, HBOT was usually scheduled at 7:30 or 9:30 a.m., while RMNES was usually performed in the afternoon, with a longer time interval, resulting in poorer synergy between the two. The specific mechanism of action needs to be explored in the future. Although both test groups 1 and 2 performed better than the control group, the clinical efficiency in this study was lower than that in previous literature, which may be related to the small sample size of this trial, its short treatment period, the overly simplified method of assessing clinical efficacy, and the nonuniform criteria.

This trial was conducted in strict accordance with the operating standards of the MNES therapy instrument, and no adverse reactions, such as seizures, skin burns at the MNES site or sympathetic excitation, as reported in previous trials, were observed during the procedure, and the whole test process was smooth.

In terms of efficacy assessment, in addition to the GCS, which is a commonly used clinical assessment scale, EEG, BAEP, and USEP were also assessed in DOC patients in this trial. Studies have shown (R. Chen et al., 1996) that although the specificity of judging the prognosis of patients with coma or vegetative state according to GCS score is high, the risk of false positive prognostic results is also high, and this phenomenon is especially common in cases of severe head injury. Therefore, the influence of subjective factors of the GCS assessment scale on the test results is reduced in combination with other scales; it also excluded the interference of the sedative‐drug sleep status of the ICU patients in the assessment of this trial, making the assessment results more objective and credible. However, a correlation analysis of the results obtained from the four assessment scales was not performed in this study.

For follow‐up, it was only possible to contact 77 patients; the mobile numbers of the families of the remaining patients were no longer valid, and they could not be contacted. Among those patients who were contacted, a better prognosis was observed in the MNES + HBOT group. Patients were discharged from the hospital with different treatment options, such as home care or transfer to the hospital for further treatment with MNES or HBOT. The prognosis at 6 months was also affected by the different treatment modalities, but MNES + HBOT was effective in accelerating the process of wakefulness, and no adverse effects were observed in the patients. Follow‐up will be continued with the patients in the future to better observe the prognostic impact of MNES + HBOT.

Due to many factors, the sample size of this trial was small, with only 120 cases included and 40 cases in each group. In addition, the efficacy of DOC resulting from different etiologies and the age of the patients were not analyzed, and cerebral perfusion was not monitored during patient treatment, including the follow‐up of long‐term effects, all of which need further in‐depth study. On the other hand, during telephone follow‐up, the nursing staff of the patient may not accurately judge the specific situation of the patient, thus affecting the follow‐up results. Moreover, the correlation between the results obtained from the four assessment scales needs to be further analyzed.

In conclusion, the results of this study showed that HBOT combined with RMNES can promote the recovery of consciousness in patients with DOC, and the effect of HBOT with simultaneous RMNES treatment on the recovery of consciousness was better than that of HBOT followed by RMNES. In addition, RMNES in the hyperbaric chamber was safe, effective, and timesaving. The use of MNES as a noninvasive rehabilitation tool has been gradually applied in clinical practice, but the problem of promoting wakefulness in DOC patients still requires joint efforts of clinical workers.

## CONFLICTOFINTEREST

The authors declare no conflict of interest.

## AUTHOR CONTRIBUTIONS

Yan‐Song Liu made substantial contributions to the conception and design. Zi‐bo Liu and Zhe Yang acquired the data and analyzed and interpreted the data. Long Zhao and Hong‐Ling Li were involved in drafting the manuscript and critically revising it for important intellectual content. Hong‐Ling Li gave final approval of the version to be published.

### PEER REVIEW

The peer review history for this article is available at: https://publons.com/publon/10.1002/brb3.2716.

## Data Availability

All data generated or analyzed during this study are included in this published article.

## References

[brb32716-bib-0001] Bennett, M. H. , Trytko, B. , & Jonker, B. (2012). Hyperbaric oxygen therapy for the adjunctive treatment of traumatic brain injury. Cochrane Database of Systematic Reviews (Online), 12, CD004609. 10.1002/14651858.CD004609 15495120

[brb32716-bib-0002] Chen, M. , Wang, J. , Wang, B. L. , Men, W. W. , Zhang, L. , & Zhang, X. N. (2019). The effects of electrically stimulating the right median nerve. Chinese Journal of Physical Medicine and Rehabilitation, 41(02), 91–95. 10.3760/cma.j.issn.0254-1424.2019.02.003

[brb32716-bib-0003] Chen, R. , Bolton, C. F. , & Young, B. (1996). Prediction of outcome in patients with anoxic coma: A clinical and electrophysiologic study. Critical Care Medicine, 24(4), 672–678. 10.1097/00003246-199604000-00020 8612421

[brb32716-bib-0004] Cooper, E. , Cooper, B. , & CHEN, J. (2006). Right median nerve electrical stimulation for the vegetative state. Nurotraumatology, 8(2), 220–224. 10.1007/4-431-28576-8_35

[brb32716-bib-0005] Cy, Y. , Wang, L. , Feng, Z. , & Shao, X. Q. (2016). Clinical and mechanistic study on the wake‐promoting effect of median nerve electrical stimulation on comatose patients after traumatic brain injury. Chinese Journal of Rehabilitation Medicine, 31(11), 1195–1199. 10.3969/j.issn.1001-1242.2016.11.004

[brb32716-bib-0006] Edlow, B. L. , Claassen, J. , Schiff, N. D. , & Greer, D. M. (2021). Recovery from disorders of consciousness: Mechanisms, prognosis and emerging therapies. Nature Reviews Neurology, 17(3), 135–156. 10.1038/s41582-020-00428-x 33318675 PMC7734616

[brb32716-bib-0007] Feng, Z. , Zhong, Y. J. , Wang, L. , & Wei, T. Q. (2015). Resuscitation therapy for traumatic brain injury‐induced coma in rats: Mechanisms of median nerve electrical stimulation. Neural Regeneration Research, 10, 594–598. 10.4103/1673-5374.155433 26170820 PMC4424752

[brb32716-bib-0008] Giacino, J. T. , Katz, D. I. , Schiff, N. D. , John, W. , Ashman, E. J. , Ashwal, S. , Barbano, R. , Hammond, F. M. , Laureys, S. , Ling, G. S. F. , Nakase‐Richardson, R. , Seel, R. T. , Yablon, S. , Getchius, T. S. D. , Gronseth, G. S. , & Armstrong, M. J. (2018). Comprehensive systematic review update summary: Disorders of consciousness: Report of the Guideline Development, Dissemination, and Implementation Subcommittee of the American Academy of Neurology; the American Congress of Rehabilitation Medicine; and the National Institute on Disability, Independent Living, and Rehabilitation Research. Archives of Physical Medicine and Rehabilitation, 99(9), 1710–1719. 10.1016/j.apmr.2018.07.002 30098792

[brb32716-bib-0009] Giacino, J. T. , Katz, D. I. , Schiff, N. D. , Whyte, J. , Ashman, E. J. , Ashwal, S. , Barbano, R. , Hammond, F. M. , Laureys, S. , Ling, G. S. F. , Nakase‐Richardson, R. , Seel, R. T. , Yablon, S. , Getchius, T. S. D. , Gronseth, G. S. , & Armstrong, M. J. (2018). Practice guideline update recommendations summary: Disorders of consciousness: Report of the Guideline Development, Dissemination, and Implementation Subcommittee of the American Academy of Neurology; the American Congress of Rehabilitation Medicine; and the National Institute on Disability, Independent Living, and Rehabilitation Research. Neurology, 91(10), 450–460. 10.1212/WNL.0000000000005926 30089618 PMC6139814

[brb32716-bib-0010] Giacino, J. T. , Shwal, S. , Childs, N. , Cranford, R. , Jennett, B. , Katz, D. I. , Kelly, J. P. , Rosenberg, J. H. , Whyte, J. , Zafonte, R. D. , & Zasler, N. D. (2002). The minimally conscious state: Definition and diagnostic criteria. Neurology, 58(3), 349–353. 10.1212/WNL.58.3.349 11839831

[brb32716-bib-0011] Gonzales‐Portillo, B. , Lippert, T. , Nguyen, H. , Lee, J. Y. , & Borlongan, C. V. (2019). Hyperbaric oxygen therapy: A new look on treating stroke and traumatic brain injury. Brain Circulation, 5(3), 101–105. 10.4103/bc.bc_31_19 31620655 PMC6785945

[brb32716-bib-0012] Greenberg, R. P. , Mayer, D. J. , Becker, D. P. , & Miller, J. D. (1977). Evaluation of brain function in severe human head trauma with multimodality evoked potentials. Part 1: Evoked brain‐injury potentials, methods, and analysis. Journal of Neurosurgery, 47(2), 150–162. 10.3171/jns.1977.47.2.0150 874542

[brb32716-bib-0013] Hermann, B. , Goudard, G. , Courcoux, V. M. , Labat, S. , Despois, L. , Bourmaleau, J. , Richard‐Gilis, L. , Faugeras, F. , Demeret, S. , Sitt, J. D. , Naccache, L. , Rohaut, B. , & Pitié‐Salpétrière hospital Neuro‐ICU . (2019). Wisdom of the caregivers: Pooling individual subjective reports to diagnose states of consciousness in brain‐injured patients, a monocentric prospective study. BMJ Open, 9(2), e026211. 10.1136/bmjopen-2018-026211 PMC6410088 30792234

[brb32716-bib-0014] Hockaday, J. M. , Potts, F. , Epstein, E. , Bonazzi, A. , & Schwab, R. S. (1965). Electroencephalographic changes in acute cerebral anoxia from cardiac or respiratory arrest. Electroencephalography and Clinical Neurophysiology, 18, 575–586. 10.1016/0013-4694(65)90075-1 14296835

[brb32716-bib-0015] Jiang, D. X. , Xie, C. , Wang, N. , Ma, D. L. , & Chen, Q. Y. (2016). Current status and shortcomings of wake‐promoting treatment. Chinese Journal of Rehabilitation, 31(3), 225–228. 10.3870/zgkf.2016.03.021

[brb32716-bib-0016] Jiang, J. Y. , Zhu, C. , Cheng, C. C. , Wang, C. H. , Lin, B. C. , & Zhu, U. X. (1988). β‐Endophin increases incerebrospinal fluid ofacute injpy patient. Chinese Journal of Neurosurgery, 4(3), 163–168. 10.13704/j.cnki.jyyx.2019.06.080

[brb32716-bib-0017] Kebapçı, A. , Dikeç, G. , & Topçu, S. (2020). Interobserver reliability of glasgow coma scale scores for intensive care unit patients. Critical Care Nurse, 40(4), e18–e26. 10.4037/ccn2020200 32737493

[brb32716-bib-0018] Li, H. D. , & Liu, Y. W. (2019). Study on the wake‐promoting effect of hyperbaric oxygen on comatose patients with heavy traumatic brain injury. Journal of PLA Preventive Medicine, 37(06), 165–166. 10.13704/j.cnki.jyyx.2019.06.080

[brb32716-bib-0019] Lin, J. W. , Tsai, J. T. , Lee, L. M. , Lin, C. M. , Hung, C. C. , Hung, K. S. , Chen, W. Y. , Wei, L. , Ko, C. P. , Su, Y. K. , & Chiu, W. T. (2008). Effect of hyperbaric oxygen on patients with traumatic brain injury. Acta Neurochirurgica. Supplement, 101, 145–149. 10.1007/978-3-211-78205-7_25 18642650

[brb32716-bib-0020] Moattari, M. , Alizadeh, S. F. , Sharifi, N. , & Zareh, N. (2016). Effects of a sensory stimulation by nurses and families on level of cognitive function, and basic cognitive sensory recovery of comatose patients with severe traumaticbrain injury: A randomized control trial. Trauma Monthly, 21(4), 3531–3541. 10.5812/traumamon.23531 PMC5282942 28180120

[brb32716-bib-0021] Morgan, M. , Lockwood, A. , Steinke, D. , Schleenbaker, R. , & Botts, S. (2012). Pharmacotherapy regimens among patients with posttraumatic stress disorder and mild traumatic brain injury. Psychiatric Services, 63(2), 182–185. 10.1176/appi.ps.201000531 22302339

[brb32716-bib-0022] Ruan, L. , Li, X. , Huang, Q. , Yi, B. , Chen, L. , Li, X. , & Gao, G. (2019). Therapeutic effect of right median nerve stimulation on NICU coma patients. Journal of Clinical Neurosurgery, 16(04), 333–335. 10.3969/j.issn.1672-7770.2019.04.011

[brb32716-bib-0023] Sankaran, R. , Radhakrishnan, K. , & Sundaram, K. R. (2019). Hyperbaric oxygen therapy in patients with hypoxic ischemic encephalopathy. Neurology India, 67(3), 728–731. 10.4103/0028-3886.263236 31347544

[brb32716-bib-0024] Shi, Y. H. , Shao, X. Q. , Feng, Z. , Zheng, C. F. , & Shuai, L. (2017). A meta‐analysis of the efficacy of median nerve electrical stimulation versus conventional therapy to promote wakefulness in comatose patients. Chinese Journal of Rehabilitation Medicine, 32(11), 1273–1277. 10.3969/j.issn.1001-1242.2017.11.014

[brb32716-bib-0025] Tal, S. , Hadanny, A. , Sasson, E. , Suzin, G. , & Efrati, S. (2017). Hyperbaric oxygen therapy can induce angiogenesis and regeneration of nerve fibers in traumatic brain injury patients. Frontiers in Human Neuroscience, 11, 508. 10.3389/fnhum.2017.00508 29097988 PMC5654341

[brb32716-bib-0026] Tao, M. , Ma, Y. , & Chen, L. (2019). Clinical observation of right median nerve stimulation on awakening in patients with massive cerebral infarction disturbance of consciousness. Chinese and Foreign Medical Research, 17(04), 135–136. 10.14033/j.cnki.cfmr.2019.04.065

[brb32716-bib-0027] Thibaut, A. , Schiff, N. , Giacino, J. , Laureys, S. , & Gosseries, O. (2019). Therapeutic interventions in patients with prolonged disorders of consciousness. Lancet Neurology, 18(6), 600–614. 10.1016/S1474-4422(19)30031-6 31003899

[brb32716-bib-0028] Tian, W. , Wang, Z. M. , & Sun, L. (2012). Clinical observation of comprehensive rehabilitation to promote awakening of persistent vegetative state. Chinese Journal of Rehabilitation, 27(4), 283–285. 10.3870/zgkf.2012.04.016

[brb32716-bib-0029] Vienne, J. , Btler, B. , Fanken, P. , & Tafti, M. (2010). Difenial effets of GABAB rceptor subypes, gamma‐hydroxybutynic acid, and baclofenon EEG activity and sleep regltion. Journal of Neuroscience, 30(42), 14194–14204. 10.1523/JNEUROSCI.3145-10.2010 20962240 PMC6634755

[brb32716-bib-0030] Wei, T. Q. , & Feng, Z. (2016). Effects of median nerve electrical stimulation on expression of γ‐aminobutyric acid b receptor in prefrontal cortex of coma rats after traumatic brain injury. Chinese Journal of Rehabilitation Medicine, 41(1), 9–13. 10.3969/j.issn.1001-1242.2016.01.003

[brb32716-bib-0031] Yang, Y. D. (2005). Relationship of handedness with language‐dominant hemisphere of patients with cerebral infarction. Chinese Journal of Tissue Engineering Research, 9(44), 157–159. 10.3321/j.issn:1673-8225.2005.44.006

[brb32716-bib-0032] Ye, D. J. , Ning, C. , Lei, Y. , & Sun, M. (2020). Time selection and course of treatment of cerebral resuscitation with hyperbaric oxygen. Chinese Critical Care Medicine, 32(2), 215–220. 10.3760/cma.j.cn121430-20200107-00040 32275009

[brb32716-bib-0033] Yokoyama, T. , Kamei, T. , & Kanno, T. (1996). Right median nerve stimulation for comatose patients. Proceedings of The Socient of Coma, 5, 117–125.

[brb32716-bib-0034] Zhang, Y. , Liu, S. , Jia, S. , & Zhang, Y. (2010). Median nerve electrical stimulation therapy for the patients with coma. Liaoning Medical Journal, 24(06), 287–288.

[brb32716-bib-0035] Zhao, K. , & Liu, A. X. (2020). Effect of right median nerve stimulation on clinical efficacy and prognosis of patients with consciousness disorder caused by traumatic brain injury. Journal of Fujian University of Traditional Chinese Medicine, 30(03), 192–196. 10.3724/SP.J.1329.2020.03005

